# Association between Ideal Cardiovascular Health Metrics and Depression in Chinese Population: A Cross-sectional Study

**DOI:** 10.1038/srep11564

**Published:** 2015-07-15

**Authors:** Zhikun Li, Xin Yang, Anxin Wang, Jing Qiu, Wei Wang, Qiaofeng Song, Xizhu Wang

**Affiliations:** 1 Department of Cardiology, Tangshan People’s Hospital, Hebei United University, Tangshan, 063000 China; 2 Department of Neurology, Beijing Tiantan Hospital, Capital Medical University, Beijing, 100050 China; 3 School of Public Health, Ningxia Medical University; 4 Beijing Municipal Key Laboratory of Clinical Epidemiology, School of Public Health, Capital Medical University, Beijing, 100050 China; 5 Postgraduate Medicine, School of Medical Sciences, Edith Cowan University, Perth, WA 6027, Australia

## Abstract

The study aimed to examine the association between ideal cardiovascular health (CVH) metrics and depression. We conducted a population-based, cross-sectional study of 6,851 participants aged 20 years or older (3,525 men and 3,326 women) living in Tangshan City, China. Information on the seven CVH metrics (including smoking, body mass index, dietary intake, physical activity, blood pressure, total cholesterol and fasting blood glucose) was collected via questionnaires, physical examination and laboratory test. Depression status was assessed using the Epidemiologic Studies Depression Scale (CES-D) and a score of 16 or above was considered depression. The relationship between CVH metrics and depression was analyzed using logistic regression. Of the 6,851 participants, 525 (7.7%) were in depression status. After adjustment for potential confounders, men in the highest quartile of ideal CVH metric summary score had a reduced likelihood of having depression compared to those in the lowest quartile (adjusted odds ratio (AOR): 0.46, 95% confidence interval (CI): 0.28–0.75, *p* = 0.002). A similar trend was found among women, even though the association was not significant (AOR = 0.74, 95%CI: 0.46–1.18, *p* = 0.211). This study suggested that better CVH status is associated with a lower risk of depression especially in Chinese male and young population.

Depression is a significant contributor to the global burden of diseases, currently affecting an estimated 350 million people worldwide. Depression is more prevalent in developing countries than developed countries. Unipolar major depression is the second largest contributor to the burden of disease in mainland China, accounting for 6.2% of the total disease burden^1^. Moderate to severe depression is a serious condition and can lead to severe consequence including suicide^2^.

Depression is a physical and psychological illness. The disease if untreated can lead to fatal consequences such as heart attacks and cerebral vascular events^3^,^4^,^5^,^6^. The best strategy to control the depression is to understand the condition and apply lifestyle modifications^7^.

American Heart Association (AHA) proposed the concept of “ideal cardiovascular health” in 2010. This refers to the simultaneous existence of three health behaviors (non-smoking, being physically active, and healthy dietary intake), and four health biomarkers (body mass index <25 kg/m^2^, untreated total cholesterol <200 mg/dl, untreated systolic/diastolic blood pressure <120/80 mmHg, and fasting plasma glucose <100 mg/dl)^8^. The “zero-level prevention” concept emphasizes the importance of healthy behaviors on preventing cardiovascular diseases.

España-Romero *et al.* reported that ideal cardiovascular factors had protective effects on depression in Caucasian populations^9^. However, within Chinese population, the prevalence of ideal cardiovascular health indicators remains unclear, and their relationships with depression have not been reported. We, therefore, conducted a cross-sectional study to explore the relationship between ideal cardiovascular metrics and depression in Chinese population.

## Results

After excluding 13 participants aged less than 20 years and 246 persons with missing information on study exposures, outcomes or important confounders, the final analyses included 6,851 persons (3,525 men and 3,326 women). Overall, 525 (7.7%) participants had depressive symptoms. Table 1 shows the characteristics of participants with or without depression. The prevalence of depression among men was comparable to that among women (8.2% vs. 7.1%, *p* = 0.086). On average, participants with depression were younger and had lower income. Regarding CVH metrics, participants with non-ideal smoking status (9.6%), physically inactive (9.8%) and having unhealthy diet (12.3%) were more likely to be depressed. There was no correlation between body mass index (BMI), total cholesterol and depression.

Table 2 shows the association between depression and cardiovascular health metric. After adjusting for age, gender, marital status, alcohol use, income level, education, history of myocardial infarction, history of stroke and cancer, and other six ideal cardiovascular metrics, we found that ideal smoking status, ideal physical activity and ideal diet intake significantly correlated with the a lower prevalence of depression (AOR = 0.78, 95%, CI: 0.61–0.99, *P* = 0.041; AOR = 0.67, 95% CI: 0.55–0.82, *P* < 0.001; and AOR = 0.43, 95% CI: 0.32–0.58, *P* < 0.001, respectively). In contrast, ideal blood pressure was associated with a higher prevalence of depression compared to those with a poor blood pressure (OR = 1.79, 95%, CI: 1.24–2.59, *P* = 0.001), but we found no significant association between BMI, total cholesterol, fasting blood glucose and depression.

Stratified analyses showed that the negative correlation of depression with ideal dietary intake and positive correlation with ideal blood pressure were consistent in men and women across different age groups. However, the negative association of depression with ideal smoking status and physical activity appeared to be more associated with men than women.

Table 3 shows the associations between depression and the summary score of ideal CVH metrics. After adjusting for potential confounders, we found that participants in the highest quartile of ideal CVH metrics summary score had a lower risk of getting depression than those in the lowest quartile of the summary score (AOR = 0.58, 95%CI: 0.43–0.78, *p* < 0.001). Stratified analyses showed that such negative association was more profound in men those whose age were below 40 years.

## Discussion

Based on this large population-based sample, the study found that participants with better CVH metric measures had a lower risk of having depression. Those in the highest quartile of the ideal CVH metric summary score had a 42% reduced odds of having depression compared to those in the lowest quartile. This negative association was stronger in men and in participants younger than 40 years.

Our research showed that ideal metrics of smoking, physical activity and dietary intake had a negative association with depression. Other studies done outside China have also come to a similar conclusion^9^,^10^. A prospective study of 5,110 Americans found that the ideal cardiovascular health components, especially healthy behaviors, were negatively correlated with depressive symptoms^9^. Likewise, a study by Ia M.K *et al.* on 30,239 participants from the United States found that depression was associated with poor metrics of CVH^10^. Health behaviors may be crucial to prevent depression and can reduce the risk of developing cardiovascular diseases. The cardiovascular health behaviors including ideal diet intake, being physically active and non-smoking are associated with a reduced likelihood of having depression^11^,^12^,^13^. Our study confirmed the findings from previous studies that ideal diet intake is associated with a lower prevalence of depression. Several studies reported that being physically inactive is associated with depression, whereas regular physical exercise can be useful to relieve insomnia and depression and improve psychological health^14^,^15^,^16^. Our study confirmed this association, even though the association appeared stronger in men. In addition, studies showed that cigarette smoking might compromise the effectiveness of treatments for depression, and increase the risk of developing depression^17^,^18^,^19^. Similar conclusion was found in our study, even though the association seems only present among men but not women (*P* *>* 0.05).

We analyzed the relationships between depression and the biomarkers indicative of ideal cardiovascular health. Previous research showed that there were negative correlation between the total cholesterol and depression^20^; however, our study disagreed with this finding. A prospective study of 5,110 Caucasian also found no significant correlation between total cholesterol and depression^9^. We did not find a correlation between fasting plasma glucose and depression, which was contrary to some previous studies^4^. There may be two possible explanations for this discrepancy. Firstly, our study may have failed to detect the association because too few participants had a poor measure of fasting blood glucose, with a proportion of 2.6% and 4.7% in participants with or without depression, respectively. Secondly, the difference may be due to ethnic difference among the participants. Participants in this study are Han-ethnic Chinese, probably having different physique and lifestyles compared with other ethnic populations.

In our study, participants with ideal blood pressure had a 79% increase in the odds of having depression compared to those with poor blood pressure. Earlier research considered low blood pressure ideal for health^21^, but more recent studies showed that low blood pressure was associated with various somatic and psychological symptoms^22^,^23^. A cross-sectional study of 60,799 participants showed that low blood pressure was associated with anxiety and depression^24^. In their study, hypotension was defined as the systolic/diastolic blood pressure less than 120/75 mmHg, which is close to the “ideal blood pressure level”. In this study, we found similar results especially in the male and young population. A study reported that neurons which controlled blood pressure by releasing neuropeptide Y may play a role in lowering blood pressure and inducing anxiety^25^.

Although our study included a large sample size and adjusted for a variety of potential confounders, several limitations should be noted. Firstly, dietary intake is defined based on questionnaire survey modified from the established food frequency questionnaire. Secondly, despite that the Center for Epidemiologic Studies Depression Scale is a validated scale and widely used for measuring depression in epidemiologic studies, the scale is not a tool for clinical diagnosis of depression. Thirdly, we do not have sufficient information on use of antidepressant medication and other treatments and thus are unable to adjust for them in the model. Fourthly, all participants in this study are from the Jidong community with relatively higher income status and attained higher education compared to the general Chinese population. Therefore, we cannot generalize our results for the entire Chinese population. Finally, this study is a cross-sectional study, which limits our ability to conclude a cause–effect relationship between ideal cardiovascular metrics and depression.

In conclusion, higher ideal cardiovascular metrics are associated with a lower prevalence of depression. Maintaining ideal cardiovascular health, i.e., non-smoking, physically active and healthy dietary intake may be of great value to prevent depression in the general population, especially among male and young Chinese population.

## Methods

### Ethics Statement

The study was conducted according to the guidelines of Helsinki Declaration and was approved by the Ethics Committee of Jidong Oilfield Inc. Medical Centers. Written informed consent was obtained from all participants.

### Study Design and Population

From July to November 2013, all residents aged 20 years and above from Jidong community were invited to participate in this study. The community is geographically located in Tangshan City, northern China and is mainly comprised of employees of the Jidong Oilfield Inc. and their family members. 7,110 residents (out of all the 9,500 residents) were willing to participate in the study and provided informed consent. Among the participants, 6,851 who had complete information on CVH metrics, depression and potential confounders were included in the analyses. At baseline, physical examinations and surveys were conducted by trained medical professionals from Medical Centers of the Jidong Oil field Inc.

### Assessment of Cardiovascular Health Metrics

Dietary intake was assessed via a brief semi-quantitative food frequency questionnaire^26^,^27^. All participants were asked the amount and frequency of the consumption of ten major food groups/items during past 12 months: vegetable, fruits, fiber-rich whole grains, eggs, red meat (beef/lamb/pork), fish/sea food, milk and dairy products, soybean products, nuts, sugar-sweetened beverage, and tea. Salt intake (gram per day) was based on self-report. Healthy dietary-intake components were defined as follows: 4.5 or more servings per day of fruits and vegetables; 2 or more servings per week of fish or shellfish; 3 or more servings per day of fiber-rich whole grains; sugary drinks once a week or less; and less than 6 gram per day of salt intake.

Body mass indexes (BMI) were defined based on measured heights (accurate to 0.1 cm) and weights (accurate to 0.1 kg), and calculated as the body weight (kg) divided by the square of height (m^2^). Blood pressure was measured using a mercury sphygmomanometer. Two readings of systolic blood pressure (SBP) and diastolic blood pressure (DBP) were taken at a five-minute interval with the participants resting in a chair during the interval. The average of the two readings was used for current analyses. If the two measurements differed by more than 5 mmHg, an additional reading was taken, and the average of the three readings was used.

Blood samples were drawn by trained phlebotomists from the participants after overnight fasting. The venous blood samples in tubes containing trisodium ethylenediaminetetraacetic acid were immediately placed on ice after antecubital venipuncture. Blood samples were then centrifuged for 10 minutes at 3,000 rotations per minute at 25 °C. After separation, plasma samples were used within four hours. Biochemical variables including total cholesterol and fasting blood glucose were measured using an autoanalyzer (Olympus, AU400, Japan) at the central laboratory in Jidong Oilfield Hospital.

According to the guidelines by America Heart Association (AHA), we defined the seven CVH metrics in three levels: “ideal”, “intermediate” and “poor”^8^. Based on the number of healthy-diet behaviors, the dietary intake metric was classified as ideal (4–5 components), intermediate (2–3 components) or poor (0–1 component). Smoking metric was classified as ideal (never or quit-smoking >12 months); intermediate (former-smoking ≤12 months) or poor (current smoking); physical activity was classified as ideal (≥150 min/week of moderate intensity or ≥75 min/week of vigorous intensity), intermediate (1–149 min/week of moderate intensity or 1–74 min/week of vigorous intensity), or poor (none). BMI was classified as ideal (<25 kg/m^2^), intermediate (25–29.9 kg/m^2^) or poor (≥30 kg/m^2^); blood pressure was classified as ideal (SBP<120 mmHg and DBP <80 mmHg and untreated), intermediate (SBP 120–139 mmHg or DBP≥80–89 mmHg or treated to goal), or poor (SBP≥140 mmHg or DBP≥90 mmHg); fasting blood glucose was classified as ideal (<100 mg/dL and untreated), intermediate (100–125 mg/dL or treated to goal), or poor (≥126 mg/dL); and total cholesterol status was classified as ideal (<200 mg/dL and untreated), intermediate (200 to 239 mg/dL or treated to goal), or poor (≥240 mg/dL).

### Assessment of depression

Depression was assessed using the Center for Epidemiologic Studies Depression Scale (CES-D). The CES-D contains 20 items and measures six components including depressed mood, feelings of guilt and worthlessness, feelings of helplessness and hopelessness, psychomotor retardation, loss of appetite and sleep disturbance. Respondents indicated how often they experienced the symptoms in last week. Their answers could be: “rarely or none of the time” (score 0), “some or little of the time” (score 1), “occasionally or a moderate amount of time” (score 2), and “most or all of the time” (score 3). Scores for the 20 items were summed to yield a total score ranging from 0 to 60, with higher scores indicating greater severity in depressive symptoms. Scores of 16 and above are considered indicative of having depressive symptoms^28^,^29^.

### Assessment of Potential Covariates

Information on demographic and clinical characteristics (age, sex, marital status, heavy alcohol consumption, personal monthly income, education and history of diseases) was collected via questionnaires. Age was classified into three categories: <40 years, 40–59 years and ≥60 years. Marital status was divided into being married and unmarried (including single, divorced or widowed). Heavy alcohol consumption was defined as a daily intake of at least 100 ml of liquor (equivalent to 240 ml of wine or 720 ml of beer) for more than a year. Previous history of disease, including myocardial infarction, stroke and cancer, was based on self-report. The average monthly income was categorized as “<¥3,000”, “¥3,000–4,999” and “≥¥5,000”. The educational attainment was categorized as “illiteracy or primary”, “middle/high school” and “college graduate or above”.

### Statistical Analyses

Continuous variables were described with mean (standard deviation, SD) and compared using ANOVA analysis. Categorical variables were described with percentages and compared using Chi-square test. Logistic regression was used to analyze the association between each CVH metric and presence of depressive symptoms by calculating the odds ratios (ORs) and 95% confidence interval (CI). We adjusted for age, gender, marital status, heavy alcohol consumption, income level, education level and history of myocardial infarction, stroke and cancer in the models because they were known as possible risk factors for depression^5^,^6^,^30^,^31^.

To assess the collective impact of ideal CVH metrics on depression, we calculated a summary score of ideal CVH metrics. Each CVH metric was assigned a score as follows: “poor” was coded as “0”, the “intermediate” was coded as “1”, and “ideal” was coded as “2”. The summary score of ideal CVH metrics for each individual was the sum of the scores of his/her seven CVH metrics. In the logistic regression models estimating the relationship between the summary score and depression, the summary was entered in the models as quartiles (with the lowest quartile as the reference).

As sensitivity analyses, we estimated the association between CVH metrics and depression stratified by sex and age groups. All statistical tests were 2-sided with the significance level set at *P* ≤ 0.05. All analyses were performed using SAS 9.3 (SAS Institute, Cary, North Carolina, USA).

## Additional Information

**How to cite this article**: Li, Z. *et al.* Association between Ideal Cardiovascular Health Metrics and Depression in Chinese Population: A Cross-sectional Study. *Sci. Rep.* **5**, 11564; doi: 10.1038/srep11564 (2015).

## Figures and Tables

**Table 1:** Characteristics of study participants by depression status.

**Characteristic**	**No Depressive SymptomsCES-D <15 (n = 6326)**	**Depressive SymptomsCES-D** ≥**16 (n = 525 )**	***p*-value**
	*Mean ± SD or N(%)*		
Age (years)	42.4 ± 13.1	38.7 ± 11.2	<0.001
Gender			0.086
Male	3236 (91.8)	289 (8.2)	
Female	3090 (92.9)	236 (7.1)	
Married			0.236
Yes	5861 (92.4)	479 (7.6)	
No	465 (91.0)	46 (9.0)	
Heavy alcohol consumption			0.625
Yes	170 (91.4)	16 (8.6)	
No	6156 (92.4)	509 (7.6)	
Income, ¥/month			0.005
<¥3000	2533 (91.4)	239 (8.6)	
¥3000–4999	3226 (92.6)	257 (7.4)	
≥¥5000	567 (95.1)	29 (4.9)	
Education level			0.162
Illiteracy/primary	230 (93.1)	17 (6.9)	
Middle school	2291 (93.1)	170 (6.9)	
College/University	3805 (91.8)	338 (8.2)	
Previous history of disease			
Myocardial infarction	35 (92.1)	3 (7.9)	0.957
Stroke	101 (91.0)	10 (9.0)	0.591
Cancer	87 (96.7)	3 (3.3)	0.120
Smoking			0.002
Ideal (never)	4702 (93.0)	355 (7.0)	
Intermediate (former)	49 (94.2)	3 (5.8)	
Poor(current smoker)	1575 (90.4)	167 (9.6)	
BMI			0.147
Ideal(<25 kg/m^2^)	3706 (91.9)	326 (8.1)	
Intermediate(25–29.99 kg/m^2^)	2149 (92.7)	170 (7.3)	
Poor (≥30 kg/m^2^)	471 (94.2)	29 (5.8)	
Physical activity			<0.001
Ideal^⊕^	3350 (94.2)	206 (5.8)	
Intermediate^☆^	553 (90.7)	57 (9.3)	
Poor (0 min/wk)	2423 (90.2)	262 (9.8)	
Diet			<0.001
Ideal (3–4 of components)	1343 (95.1)	69 (4.9)	
Intermediate(2 of components)	3636 (93.1)	268 (6.9)	
Poor (0–1 of components)	1347 (87.7)	188 (12.2)	
Total cholesterol			0.332
Ideal (<200 mg/dl)	5028 (92.1)	431 (7.9)	
Intermediate (200–239 mg/dl)	1085 (93.4)	77 (6.6)	
Poor (≥240 mg/dl)*	213 (92.6)	17 (7.4)	
Blood pressure			<0.001
Ideal (<120/80 mmHg)	2310 (90.6)	240 (9.4)	
Intermediate (SBP129–139 or DBP80–90 mmHg)	3083 (92.8)	239 (7.2)	
Poor (SBP ≥ 140 or DBP > 90 mmHg)†	933 (95.3)	46 (4.7)	
Fasting blood glucose			0.009
Ideal (<100 mg/dl)	5053 (91.9)	447 (8.1)	
Intermediate (100–125 mg/dl)	976 (93.8)	64 (6.1)	
Poor (≥126 mg/dl)‡	297 (95.5)	14 (4.5)	

CES-D = Center for Epidemiologic Studies Depression Scale ( ≥16 score as depressive symptom); BMI = body mass index; DBP = diastolic blood pressure; SBP = systolic blood pressure.

^⊕^Defined as ≥150 min/wk moderate intensity or ≥75 min/wk vigorous intensity or ≥150 min/wk moderate + vigorous.

^☆^Defined as ≥1–149 min/wk moderate intensity or 1–74 min/wk vigorous intensity or 1–149 min/wk moderate + vigorous.

^*^Plus no previous physician diagnosis of hypercholesterolemia.

^†^Plus no previous physician diagnosis of hypertension.

^‡^Plus no previous physician diagnosis of diabetes or no use of insulin.

**Table 2:** Associations of depression status with each component of cardiovascular health metric, adjusted odds ratio * (95% confidence interval).

**Metrics**	**Total**	**Gender**		**Age (years)**		
		**Male**	**Female**	**<40**	**40–59**	≥**60**
Smoking						
Intermediate	0.75 (0.23–2.47)	0.77 (0.23–2.55)	※	0.48 (0.06–3.79)	0.55 (0.07–4.29)	4.34 (0.37–50.99)
Ideal	0.78 (0.61–0.99)	0.73 (0.57–0.95)	1.61 (0.43–6.01)	0.82 (0.60–1.12)	0.74 (0.47–1.16)	1.11 (0.31–3.99)
BMI						
Intermediate	1.27 (0.84–1.93)	1.14 (0.70–1.85)	1.71 (0.75–3.91)	0.92 (0.57–1.49)	3.49 (1.06–11.41)	3.32 (0.33–33.33)
Ideal	1.24 (0.81–1.88)	1.16 (0.71–1.91)	1.51 (0.68–3.48)	0.80 (0.49–1.29)	3.88 (1.18–12.74)	4.18 (0.41–41.86)
Physical activity						
Intermediate	0.98 (0.72–1.33)	0.99 (0.67–1.47)	0.90 (0.54–1.49)	0.87 (0.59–1.28)	1.01 (0.57–1.78)	3.06 (0.57–16.44)
Ideal	0.67 (0.55–0.82)	0.56 (043–0.73)	0.80 (0.59–1.49)	0.60 (0.46–0.78)	0.79 (0.56–1.10)	0.65 (0.28–1.53)
Diet						
Intermediate	0.60 (0.49–0.74)	0.62 (0.47–0.81)	0.58 (0.42–0.80)	0.60 (0.46–0.77)	0.51 (0.35–0.74)	0.83 (0.32–2.19)
Ideal	0.43 (0.32–0.58)	0.46 (0.30–0.71)	0.41 (0.27–0.63)	0.44 (0.30–0.66)	0.37 (0.23–0.61)	0.19 (0.03–1.02)
Total cholesterol						
Intermediate	0.76 (0.43–1.32)	0.83 (0.36–1.94)	0.72 (0.34–1.54)	0.50 (0.18–1.34)	1.31 (0.56–3.03)	0.40 (0.10–1.58)
Ideal	0.73 (0.43–1.23)	0.85 (0.38–1.89)	0.67 (0.33–1.35)	0.59 (0.23–1.49)	1.04 (0.46–2.32)	0.36 (0.10–1.27)
Blood pressure						
Intermediate	1.41 (1.01–1.97)	1.44 (0.96–2.15)	1.31 (0.70–2.43)	1.89 (1.09–3.25)	0.99 (0.61–1.62)	1.45 (0.49–4.25)
Ideal	1.79 (1.24–2.59)	1.78 (1.13–2.81)	1.79 (0.94–3.42)	2.27 (1.27–4.05)	1.50 (0.88–2.54)	3.51 (0.97–12.75)
Fasting blood glucose						
Intermediate	1.24 (0.68–2.27)	1.30 (0.59–2.89)	1.10 (0.43–2.78)	0.80 (0.25–2.51)	1.20 (0.50–2.86)	1.57 (0.39–6.32)
Ideal	1.30 (0.73–2.30)	1.41 (0.66–3.00)	1.08 (0.45–2.61)	0.77 (0.26–2.28)	1.55 (0.69–3.48)	0.64 (0.15–2.65)

^*^The reference group includes patients with poor metric of cardiovascular health. The following potential confounders were adjusted for each OR: sex, age, marital status, heavy alcohol consumption, income level, education level, previous history of myocardial infarction, stroke, cancer and the other six cardiovascular health metrics.

※None of the women were former smokers but have quit smoking for ≤12 months.

**Table 3:** Associations of depression status with score of ideal Cardiovascular Health Metrics by age and gender, adjusted odds ratio * (95% confidence interval).

**Metrics**	**Prevalence of depression (%)**	**Total**	**Gender**		**Age (years)**		
			**Male**	**Female**	**<40**	**40–59**	≥**60**
Quartile 1 (≤8)	2.31	1.00	1.00	1.00	1.00	1.00	1.00
Quartile 2 (9–10)	2.71	0.94 (0.75–1.19)	0.92 (0.70–1.21)	1.06 (0.67–1.68)	0.89 (0.66–1.20)	0.96 (0.64–1.44)	1.41 (0.59–3.38)
Quartile 3 (11)	1.21	0.67 (0.50–0.91)	0.63 (0.41–0.96)	0.81 (0.49–1.34)	0.56 (0.38–0.82)	0.97 (0.58–1.61)	0.24 (0.03–1.99)
Quartile 4 (12–14)	1.43	0.58 (0.43–0.78)	0.46 (0.28–0.75)	0.74 (0.46–1.18)	0.43 (0.29–0.64)	0.90 (0.56–1.45)	0.75 (0.19–2.90)

^*^The following potential confounders were adjusted for each OR: sex, age, marital status, heavy alcohol consumption, income level, education level, previous history of myocardial infarction, stroke, or cancer.
